# Supplementary material for: Iterative improvement in the automatic modular design of robot swarms
Source: PeerJ Comput Sci. 2020 Dec 7;6:e322. doi: 10.7717/peerj-cs.322 (PMC7924708; doi:10.7717/peerj-cs.322)
Supplement: Supplemental Information 3 [file peerj-cs-06-322-s003.zip › argos3/doc/api/standalone/a00648_source.html]

ARGoS: plugins/simulator/visualizations/qt-opengl/qtopengl\_lua\_statetree\_item.cpp Source File


- Main Page
- Related Pages
- Namespaces
- Classes
- Files

- File List
- File Members

# plugins/simulator/visualizations/qt-opengl/qtopengl\_lua\_statetree\_item.cpp

Go to the documentation of this file.

```
00001 
00007 #include "qtopengl_lua_statetree_item.h"
00008 #include <cstdio>
00009 
00010 namespace argos {
00011 
00012    /****************************************/
00013    /****************************************/
00014 
00015    CQTOpenGLLuaStateTreeItem::CQTOpenGLLuaStateTreeItem(CQTOpenGLLuaStateTreeItem* pc_parent) :
00016       m_pcParent(pc_parent) {}
00017 
00018    /****************************************/
00019    /****************************************/
00020 
00021    CQTOpenGLLuaStateTreeItem::CQTOpenGLLuaStateTreeItem(QList<QVariant>& list_data,
00022                                                         CQTOpenGLLuaStateTreeItem* pc_parent) :
00023       m_listData(list_data),
00024       m_pcParent(pc_parent) {}
00025 
00026    /****************************************/
00027    /****************************************/
00028 
00029    CQTOpenGLLuaStateTreeItem::~CQTOpenGLLuaStateTreeItem() {
00030       qDeleteAll(m_listChildren);
00031    }
00032 
00033    /****************************************/
00034    /****************************************/
00035 
00036    CQTOpenGLLuaStateTreeItem* CQTOpenGLLuaStateTreeItem::GetParent() {
00037       return m_pcParent;
00038    }
00039 
00040    /****************************************/
00041    /****************************************/
00042 
00043    CQTOpenGLLuaStateTreeItem* CQTOpenGLLuaStateTreeItem::GetChild(size_t un_idx) {
00044       return m_listChildren.value(un_idx);
00045    }
00046 
00047    /****************************************/
00048    /****************************************/
00049 
00050    void CQTOpenGLLuaStateTreeItem::AddChild(CQTOpenGLLuaStateTreeItem* pc_child) {
00051       m_listChildren.append(pc_child);
00052    }
00053 
00054    /****************************************/
00055    /****************************************/
00056 
00057    void CQTOpenGLLuaStateTreeItem::RemoveChild(CQTOpenGLLuaStateTreeItem* pc_child) {
00058       m_listChildren.removeOne(pc_child);
00059    }
00060 
00061    /****************************************/
00062    /****************************************/
00063 
00064    size_t CQTOpenGLLuaStateTreeItem::GetNumChildren() const {
00065       return m_listChildren.count();
00066    }
00067 
00068    /****************************************/
00069    /****************************************/
00070 
00071    bool ItemLessThan(const CQTOpenGLLuaStateTreeItem* pc_i1,
00072                      const CQTOpenGLLuaStateTreeItem* pc_i2) {
00073       if(pc_i1->GetData(0).type() == QVariant::Double &&
00074          pc_i2->GetData(0).type() == QVariant::Double) {
00075          return pc_i1->GetData(0).toDouble() < pc_i2->GetData(0).toDouble();
00076       }
00077       else {
00078          return pc_i1->GetData(0).toString().toLower() < pc_i2->GetData(0).toString().toLower();
00079       }
00080    }
00081 
00082    void CQTOpenGLLuaStateTreeItem::SortChildren() {
00083       qSort(m_listChildren.begin(), m_listChildren.end(), ItemLessThan);
00084       foreach(CQTOpenGLLuaStateTreeItem* pcItem, m_listChildren) {
00085          pcItem->SortChildren();
00086       }
00087    }
00088 
00089    /****************************************/
00090    /****************************************/
00091 
00092    QVariant CQTOpenGLLuaStateTreeItem::GetData(int n_col) const {
00093       return m_listData.value(n_col);
00094    }
00095 
00096    /****************************************/
00097    /****************************************/
00098 
00099    int CQTOpenGLLuaStateTreeItem::GetRow() {
00100       if(m_pcParent != NULL) {
00101          return m_pcParent->m_listChildren.indexOf(const_cast<CQTOpenGLLuaStateTreeItem*>(this));
00102       }
00103       else {
00104          return 0;
00105       }
00106    }
00107 
00108    /****************************************/
00109    /****************************************/
00110 
00111 }
```

---

Generated on 10 Jul 2018 for ARGoS by 
 1.6.1
